# Performance of Youth Athletes Is Not Consistently Determined by Maturity or Training Experience: A Cross-Sectional Study

**DOI:** 10.3390/jfmk11020166

**Published:** 2026-04-22

**Authors:** Anastasios Lykidis, Rafail Georgios Pechlivanos, Anthi Angelou, Nikolaos Varvariotis, Chrysostomos Sahinis, Ioannis G. Amiridis, Roger M. Enoka

**Affiliations:** 1 Laboratory of Neuromechanics, Department of Physical Education and Sport Sciences at Serres, Aristotle University of Thessaloniki, 62500 Serres, Greecerapechli@phed-sr.auth.gr (R.G.P.); anthiangelo@phed-sr.auth.gr (A.A.); nsvarvar@phed-sr.auth.gr (N.V.); sachinisc@phed-sr.auth.gr (C.S.); 2 Department of Integrative Physiology, University of Colorado, Boulder, CO 80309, USA

**Keywords:** maturation, training exposure, puberty, neuromuscular function

## Abstract

**Objective:** The purpose of this study was to compare the influence of biological maturity status and training experience on motor performance in young athletes of different sport disciplines. **Methods:** Youth athletes (n = 84, 23 females) from five different sports (basketball, volleyball, track and field, wrestling, and badminton) participated in this study. Jump height was measured for the squat jump (SJ) and countermovement jump (CMJ). Peak torque during maximal voluntary contractions (MVCs) and torque steadiness at 20% MVC were assessed during plantar flexion (PF) and dorsiflexion (DF). Postural control was assessed with the one-leg test for both the right and left legs. K-means clustered analysis categorized participants into groups of low and high performers. **Results:** High performers had greater training experience than low performers for the SJ (*p* < 0.05), with no difference in maturity status (*p* > 0.05). Similarly, high performers had greater training experience (*p* < 0.05) than low performers for the CMJ, with no difference in maturity status (*p* > 0.05). High performers were more mature than low performers for MVC torque of DF (*p* < 0.001) and PF (*p* < 0.001), with no group differences in training experience (DF: *p* > 0.05; PF: *p* > 0.05). Maturity status for torque steadiness differed only for DF (*p* < 0.001), whereas there was no difference for PF (*p* > 0.05). There were no differences in either maturity status or training experience for one-leg-stance time (*p* > 0.05). **Conclusions:** These findings suggest that maturity status and training experience are linked to performance, although their relative roles differ across tasks. These findings reflect an interaction between biological maturity, training background and sports performance in youth athletes.

## 1. Introduction

Participation of children in sports is typically organized according to chronological age categories. Although this classification facilitates organizational structure, it often leads to performance differences among individuals who differ in biological age, as the same chronological age may correspond to differences in maturity status [1,2,3]. Furthermore, performance is modified by training experience, particularly in team sports where tactical, technical, and physical abilities are integrated during competition [4,5].

In sports, maturity status is often used in talent identification by coaches and researchers attempting to recognize “more mature” individuals as those more likely to achieve higher performance scores, such as those involved in sprinting and jumping [6]. This approach is based on the premise that individuals who are past peak height velocity may demonstrate superior performance due to maturation-related anatomical and neural adaptations [7]. For instance, boys who are past peak height velocity demonstrate greater muscle thickness, pennation angle, and fascicle length in the gastrocnemius medialis and vastus lateralis, which likely enhance sprinting and jumping performance [8]. Additionally, neural adaptations during maturation appear to influence youth athletic performance, including altered coactivation between agonist and antagonist muscles, differences in motor unit activity, and a reduced ability to achieve adult-like muscle activation rates [9,10]. Nevertheless, the association between maturity and performance likely differs across tasks, especially those requiring coordinated actions [11].

In addition to maturation status, training experience modifies selected features of athletic performance. Engagement in formal training, for example, promotes the parallel development of physical capabilities and techniques that are specific to the targeted sport. For example, participation in sports training has been shown to improve jumping performance in athletes around puberty, whereas similar improvements are not consistently observed in older individuals [12]. Similarly, increases in jump height have been reported in active youth athletes compared with a control group who were matched for chronological age, which indicates that adaptations elicited by team-sports training, such as sprinting and jumping, are a sufficient training stimulus to improve jump height [13]. Consequently, both maturation status and training experience can enhance the performance of youth athletes by improving muscle strength, jumping, and balance.

However, it remains unclear whether high-performing athletes are characterized by greater biological maturity or training experience. Stratification of athletes based on performance metrics—rather than the conventional approach of comparing cohorts that differ in maturity status and training experience—allows for direct assessment of these attributes. The purpose of this study was to compare the biological maturity status and training experience of high- and low-performing youth athletes. We hypothesized that biological maturity and training experience would exert separate and independent effects on the performance capabilities of youth athletes.

## 2. Materials and Methods

### 2.1. Participants

Participants were recruited via convenience sampling. Enrollment was voluntary, and written informed consent was obtained from all individuals and their legal guardians prior to beginning the study. A familiarization session was conducted one week prior to the experimental session at the same time of day. Healthy athletes (n = 84, 23 females and 61 males; age: 13.3 ± 1.7 years, body mass: 57 ± 14 kg, height: 165 ± 11 cm) from five different sports (basketball = 51, volleyball = 12, track and field = 8, wrestling = 7, and badminton = 6) participated in the study (Table 1). Participants had no history of musculoskeletal injuries during the preceding six months, no neurological disorders, and had been engaged in organized sports for at least two consecutive years. Experimental procedures were approved by the Aristotle University of Thessaloniki Ethics Committee in accordance with the Declaration of Helsinki (ERC-023/2025).

### 2.2. Maturity Status Calculation and Training Experience

Maturity offset was calculated from chronological age and anthropometric measures, and adjusted for sex-specific differences as previously proposed [14]. Regression equations for males and females were:

Maturity Offset_males_ = −9.236 + 0.0002708 × (leg length × sitting height) − 0.001663 × (age × leg length) + 0.007216 × (age × sitting height) + 0.02292 × (weight/height × 100)(1)

Maturity Offset_females_ = −9.376 + 0.0001882 × (leg length × sitting height) + 0.0022 × (age × leg length) + 0.005841 × (age × sitting height) − 0.002658 × (age × weight) + 0.07693 × (weight/height × 100)(2)

The dependent variable (Maturity Offset) refers to the years from peak height velocity. Training experience was reported as the number of years since the athlete’s first formal engagement in the sport, with a minimum of 1.5 consecutive years of training with an average of ≥3 training sessions per week.

### 2.3. Jumping Performance

Jumping ability was evaluated with SJ and CMJ performed on a force-plate (1000 Hz, type 9253B, Kistler Instruments Ltd., Winterthur, Switzerland). Participants began the SJ from a squat position with knees flexed at 90° and performed a single upward movement as fast as possible, without any preliminary downward movement. The CMJ began from an upright standing position, followed by a downward movement to 90° knee flexion and then an immediate, rapid upward movement to achieve maximal jump height. Participants were instructed to keep their hands on their hips during both jumps. Each jump was performed at least twice, with a ≤5% difference between trials, and the highest jump height was used in the analysis. Test–retest reliability within a trial was high for the two jumps [Intraclass correlation coefficient: 0.98 (95% CI: 0.94–0.99), standard error of measurement: 1.24% (95% CI: 0.5–2.1%), and Coefficient of Variation: 2.55 (95% CI: 1.12–3.4%)].

Jump height (m) was estimated from take-off velocity using the equation h = V_takeoff_^2^/2g, where h represents jump height in meters and g is the acceleration due to gravity (9.81 m·s^−2^). V_takeoff_ was estimated from the vertical ground reaction force as measured with MARS software (version 5.0.0.149) at 1000 Hz. Because each jump began from a stationary position, V_takeoff_ was equal to the net impulse of the vertical component of the ground reaction force relative to the mass of the individual. Impulse was obtained by numerically integrating (trapezoidal method) the net force–time curve during the propulsion phase, where the net vertical force was calculated by subtracting body weight from the measured vertical ground reaction force. The onset of the propulsion phase was defined as the point at which vertical force exceeded body weight, and take-off was identified as the instant when vertical ground reaction force fell below 20 N.

### 2.4. Torque Steadiness

Participants were seated on an adjustable chair with the right leg secured to an isometric dynamometer (TF022-NEG1, OT Bioelettronica, Turin, Italy). The ankle joint was positioned at 110°, knee joint was fully extended (180°), hip joint was at 120°, and the back was supported. After completing a standardized warm-up routine with the plantar flexor and dorsiflexor muscles, participants rested for 5 min [15]. Subsequently, a monitor was positioned ~1.5 m in front of the chair at eye level to provide visual feedback of target and applied torques during each trial.

Peak torques during maximal voluntary contractions (MVCs) were determined during dorsiflexion and plantar flexion from at least two recorded trials with a ≤±5% difference between trials [16]. Torque steadiness was assessed during submaximal isometric contractions at 20% of MVC, using a trapezoidal force profile with a 4 s ramps up and down and a 20 s steady plateau. Participants were instructed to keep the applied torque as steady as possible and to avoid any corrective actions. The applied torque was visualized as a blue line, the target trajectory as a light blue line, and the permissible error margins (5%) as red lines. The intensity and duration of contractions were based on established protocols [17,18]. The force signal recorded during plantar flexion and dorsiflexion was converted to ankle joint torque using the external moment arm of the dynamometer relative to the ankle joint axis. Maximal isometric torque (N·m) during plantar flexion and dorsiflexion was quantified as the peak torque computed with a 500 ms moving-average window. Torque steadiness was quantified over a 6 s interval during the plateau phase of the trapezoidal contraction by calculating Coefficient of Variation (CoV; standard deviation/mean torque × 100) with a custom-written script on MATLAB R2023b.

### 2.5. Postural Control

Postural control during quiet standing was evaluated when participants stood barefoot on a force platform (1000 Hz, type 9253B, Kistler Instruments Ltd., Winterthur, Switzerland) during one-leg stance. Each leg performed a 10 s trial [19,20]. The stance leg faced forwards, and the contralateral leg was lifted off the ground with the hip flexed to approximately 45° (0° = standing position) and the knee flexed to approximately 45° (0° = full extension). The eyes focused on a visual marker (diameter: 3 cm) that was positioned 1.5 m away at eye level. Each leg was assessed at least twice, and the center-of-pressure velocity (CoPvel Fort Worth, TX, USA) in the x-y plane (forward-backward and side-to-side directions) was calculated. The lowest value (mm/s) was retained for analysis.

### 2.6. Statistics

Statistical analyses were conducted in SPSS Statistics (version 29.0.2). All performance variables are reported as mean ± standard deviation (SD). Normality of all variables was assessed separately using the Shapiro–Wilk test. Subsequently, individual clustering analyses were performed for each variable using the k-means method to classify participants into two groups, labeled as “Low” and “High” performers. To ensure that the selected number of clusters was appropriate, cluster validity was evaluated using the silhouette coefficient. This analysis supported a two-cluster solution, indicating adequate separation and cohesion between clusters. Independent samples *t*-tests were then conducted to examine differences between the two clusters in terms of maturity status and training experience for each variable. Effect sizes were calculated using Cohen’s d and interpreted as small (0.2), moderate (0.5), large (0.8), or very large (>1.3). Ninety-five percent confidence intervals (95% CI) were calculated for all mean differences. Statistical significance was set at *p* < 0.05.

## 3. Results

### 3.1. Jump Height

**Figure 1:**
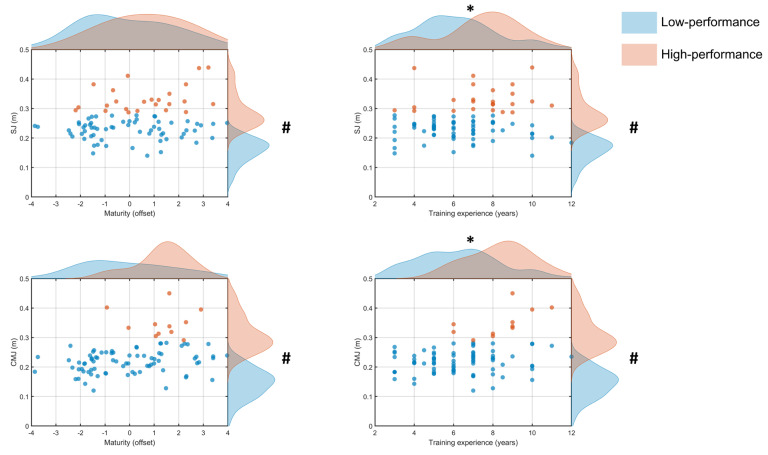
Individual squat jump (SJ; top panels) and countermovement jump (CMJ; bottom panels) heights (m) plotted against maturity offset (left panels) and training experience (right panels) for the low (blue) and high (orange) performers. Points represent individual participants; shaded marginal density curves illustrate the distribution of SJ/CMJ values (right) and the corresponding predictor variable (top) for each group. # *p* < 0.05 significant differences between clusters in jumping performance. * *p* < 0.05 significant differences between clusters in training experience.

Cluster analysis for SJ height showed that 22 of 84 (26%) participants were classified as High performers (0.34 ± 0.05 m) and 62 (74%) were identified as Low performers (0.23 ± 0.03 m), with a significant difference between clusters confirmed by ANOVA [F_(1,82)_ = 139.7, *p* < 0.001]. Independent *t*-tests revealed no significant difference [t_(82)_ = 1.2, *p* > 0.05, d = 0.3] in maturity status between Low (−0.05 ± 1.8 yrs, 95% CI [−2.6, 2.5]) and High (0.5 ± 1.9 yrs, 95% CI [−0.3, 1.3]) performers (Figure 1). Conversely, training experience was significantly greater [t_(82)_ = 2.3, *p* < 0.05, d = 0.8] for the High performers (7.9 ± 2.2 yrs, 95% CI [−5.6, 6.7]) than the Low performers (6.2 ± 2.0 yrs, 95% CI [−6.9, 8.8]).

The two groups for CMJ height comprised 11 of 84 (13%) participants as High performers (0.35 ± 0.05 m) and 73 (87%) as Low performers (0.22 ± 0.04 m) with a significant difference between clusters [F(_1,82)_ = 109.6, *p* < 0.001]. There was no significant difference [*t*_(82)_ = 0.2, *p* > 0.05, d = 0.07] in maturity status (Figure 1) between High (0.2 ± 1.8 yrs, 95% CI [−1.0, 1.4]) and Low (0.07 ± 1.9 yrs, 95% CI [−0.4, 0.5]) performers. Training experience (Figure 1) also differed significantly [t_(82)_ = 3.1, *p* < 0.05, d = 1.02], with greater values for the High performers (8.4 ± 1.6 yrs, 95% CI [7.3, 9.4]) than the Low performers (6.3 ± 2.2 yrs, 95% CI [5.8, 6.8]).

### 3.2. Maximal Voluntary Contraction Torque

**Figure 2:**
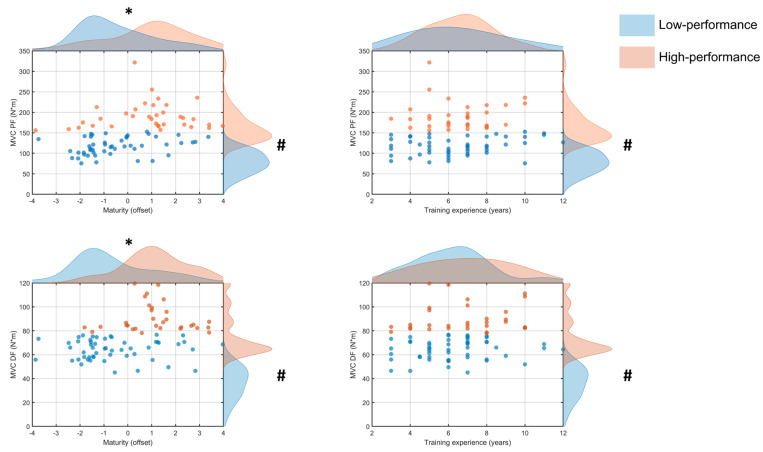
Peak MVC torque (N·m) for plantar flexors (PF; top panels) and dorsiflexors (DF; bottom panels) plotted against maturity offset (left panels) and training experience (right panels) for the low (blue) and high (orange) performers. Points represent individual participants; shaded marginal density curves illustrate the distribution of maximal torque values (right) and the corresponding predictor variable (top) for each group. # *p* < 0.05 significant differences between clusters in maximal torque. * *p* < 0.05 significant differences between clusters in maturity status.

Cluster analysis of MVC torque for the plantar flexors indicated that 35 of 84 (42%) participants were classified as High performers (191 ± 34 N·m) and 49 (58%) as Low performers (117 ± 21 N·m) with a significant difference between clusters [F_(1,82)_ = 148.3, *p* < 0.001]. Maturity status was significantly greater [t_(82)_ = 3.5, *p* < 0.001, d = 0.8] for the High (0.8 ± 1.8 yrs, 95% CI [0.2, 1.4]) than the Low performers (−0.5 ± 1.6 yrs, 95% CI [−0.9, 0.0]) (Figure 2). There was no significant difference in training experience [*t*_(82)_ = 0.2, *p* > 0.05, d = 0.0] observed between Low (6.5 ± 2.4 yrs, 95% CI [5.8, 7.1]) and High performers (6.6 ± 2.1 yrs, 95% CI [6.0, 7.2]) (Figure 2).

Cluster analysis of MVC torque for the dorsiflexors distinguished 31 of 84 (37%) participants as High performers (90 ± 12 N·m), and 53 (63%) as Low performers (64 ± 9 N·m) with a significant difference between clusters [F_(1,82)_ = 136.0, *p* < 0.001]. Maturity status was significantly greater [t(82) = 4.8, *p* < 0.001, d = 1.0] for the High (1.1 ± 1.3 yrs, 95% CI [0.6, 1.6]) than the Low (−0.6 ± 1.7 yrs, 95% CI [−1.0, −0.1]) performers (Figure 2). Training experience did not differ significantly [*t*_(82)_ = 1.2, *p* > 0.05, d = 0.3] between the Low (6.3 ± 2.1 yrs, 95% CI [5.7, 6.9]) and High performers (6.9 ± 2.2 yrs, 95% CI [6.2, 7.5]) (Figure 2).

### 3.3. Torque Steadiness

**Figure 3:**
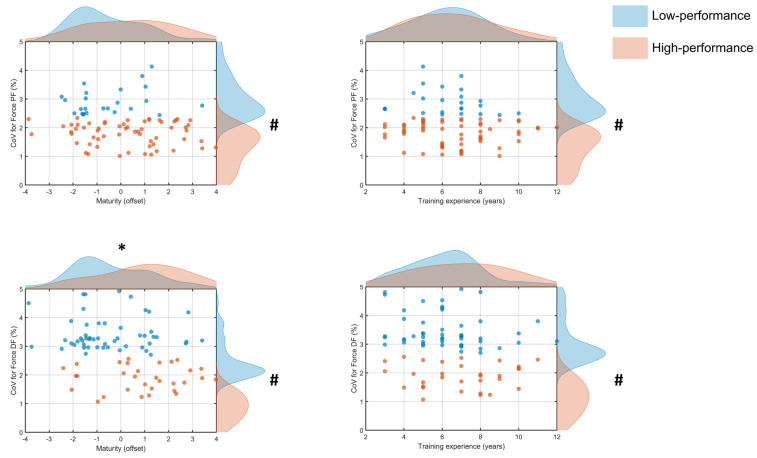
Coefficient of Variation (CoV) for torque (%) for plantar flexors (PF; top panels) and dorsiflexors (DF; bottom panels) plotted against maturity offset (left panels) and training experience (right panels) for the low (blue) and high (orange) performers. Points represent individual participants; shaded marginal density curves illustrate the distribution of maximal torque values (right) and the corresponding predictor variable (top) for each group. # *p* < 0.05 significant differences between clusters in torque steadiness. * *p* < 0.05 significant differences between clusters in maturity status.

Analysis of the CoV for plantar flexors torque indicated that 60 of 84 (71%) participants were classified as High performers (1.8 ± 0.4%) and 24 (29%) as Low performers (2.9 ± 0.5%) with a significant difference between clusters [F_(1,82)_ = 120.6, *p* < 0.001]. There was no significant difference in maturity status [t_(82)_ = 1.9, *p* > 0.05, d = 0.5] between the Low (0.3 ± 1.8 yrs, 95% CI [−0.5, 1.1]) and High performers (−0.5 ± 1.5 yrs, 95% CI [−0.9, −0.1]) (Figure 3). Similarly, training experience did not differ [t_(82)_ = 0.8, *p* > 0.05, d = 0.2] between Low (6.2 ± 1.8 yrs, 95% CI [5.5, 7.0]) and High performers (6.6 ± 2.2 yrs, 95% CI [6.0, 7.2]) (Figure 3).

A similar analysis of the CoV for dorsiflexion torque identified 31 of 84 (37%) participants as High performers (1.9 ± 0.4%) and 53 (63%) as Low performers (3.4 ± 0.6%) with a significant difference between clusters [F_(1,82)_ = 161.5 *p* < 0.001]. Maturity status was significantly greater [t_(82)_ = 3.4, *p* < 0.001, d = 0.8] for the High (0.9 ± 1.7 yrs, 95% CI [0.3, 1.5]) than the Low performers (−0.4 ± 1.6 yrs, 95% CI [−0.8, 0.04]) (Figure 3). Conversely, there was no significant difference [t_(82)_ = 1.3, *p* > 0.05, d = 0.4] in training experience between the Low (6.2 ± 2.0 yrs, 95% CI [5.7, 6.8]) and High performers (7.0 ± 2.2 yrs, 95% CI [6.1, 7.8]) (Figure 3).

### 3.4. Postural Control

**Figure 4:**
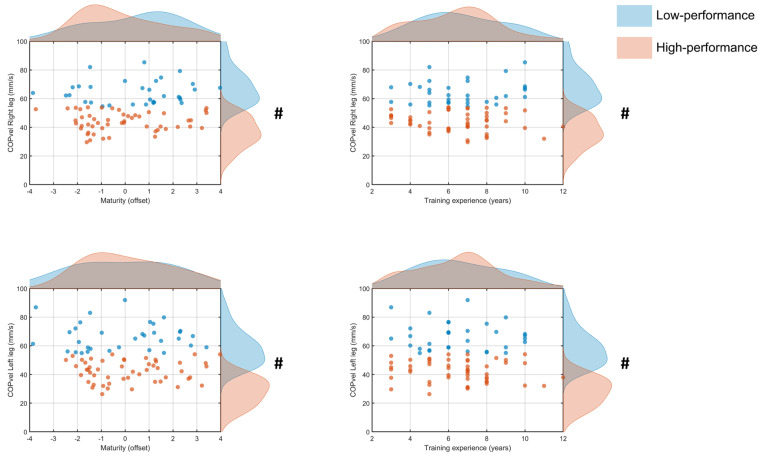
Center-of-pressure velocity (COPvel) values (mm/s) for right (top panels) and left (bottom panels) legs plotted against maturity offset (left panels) and training experience (right panels) for the low (blue) and high (orange) performers. Points represent individual participants; shaded marginal density curves illustrate the distribution of maximal torque values (right) and the corresponding predictor variable (top) for each group. # *p* < 0.05 significant differences between clusters in COPvel.

Analysis of the COPvel for one-leg stance on the right leg resulted in 52 of 84 (62%) participants being categorized as High performers (43.9 ± 6.7 mm/s) and 32 (38%) as Low performers (64.5 ± 8.0 mm/s) with a significant difference between clusters [F_(1,82)_ = 146.6, *p* < 0.001]. There was no significant difference [t_(82)_ = 1.3, *p* > 0.05, d = 0.3] for maturity status between Low (0.4 ± 1.9 yrs, 95% CI [−0.3, 1.1]) and High performers (−0.1 ± 1.8 yrs, 95% CI [−0.6, 0.4]) (Figure 4). Also, training experience did not differ [t_(82)_ = 0.5, *p* > 0.05, d = 0.1] between Low (6.7 ± 2.1 yrs, 95% CI [5.9, 7.4]) and High performers (6.4 ± 2.2 yrs, 95% CI [5.8, 7.0]) (Figure 4).

Similarly, the COPvel for one-leg stance on the left leg resulted in 51 of 84 (61%) being classified as High performers (42.3 ± 7.5 mm/s) and 33 (39%) as Low performers (66.5 ± 9.7 mm/s) with a significant difference [F_(1,82)_ = 165.2, *p* < 0.001]. There was no significant difference [t_(82)_ = 0.8, *p* > 0.05, d = 0.2] in maturity status between Low (−0.10 ± 2.0 yrs, 95% CI [−0.8, 0.6]) and High performers (0.21 ± 1.7 yrs, 95% CI [−0.3, 0.7]) (Figure 4). Also, training experience did not differ [t_(82)_ = 0.1, *p* > 0.05, d = 0.1] between Low (6.6 ± 2.1 yrs, 95% CI [5.8, 7.3]) and High performers (6.5 ± 2.2 yrs, 95% CI [5.9, 7.1]) (Figure 4).

## 4. Discussion

This study compared maturity offset and training experience between youth athletes classified as high or low performers across four tasks: vertical jump, muscle strength, torque steadiness, and postural control. Maturity offset differed between the two performance groups for MVC torque for the dorsiflexors and plantar flexors, and torque steadiness for the dorsiflexors, whereas training experience differed only for the two jump heights (SJ and CMJ). In contrast, there was no significant difference due to maturity offset or training experience between the two performance groups for either plantar flexor torque steadiness or one-leg stance.

### 4.1. Influence of Maturity on Strength, Power, and Torque Control

Athletes classified as high performers for maximal torque (Figure 2) for the dorsiflexor and plantar flexors were generally more advanced in maturity status. A similar pattern was observed for torque steadiness during dorsiflexion (Figure 3). These findings are consistent with the reported associations between maturation and neuromuscular function, whereby progression around and after peak height velocity is often accompanied by greater muscle size and force-generating capacity [5,7,21]. Several physiological processes may explain these observations. For instance, as children mature, muscle thickness and physiological cross-sectional area increase, whereas tendons become stiffer [8,22]. These morphological adaptations could enhance maximal torque and improve the storage and return of elastic energy during the stretch–shorten cycle, which enhances performance. These changes also appear to be accompanied by neural adaptations that further augment performance [23,24,25]. Less mature individuals exhibit greater coactivation and less effective pre-activation strategies, which can impair rapid force production and torque control [7,26].

### 4.2. Specificity of Training Experience

Training experience differentiated performance between groups only for jump height (SJ and CMJ; Figure 1). The distribution of high performers was similar for SJ (45% basketball, 27% volleyball, 14% wrestling and badminton) and CMJ (36% basketball and volleyball, 14% wrestling, 18% badminton), suggesting that greater training exposure is linked to better jumping performance in sports with frequent high-intensity lower limb actions. This is plausible given the repeated intense demands of these sports. For example, basketball players perform ~50 jumps during a competitive game [27,28], whereas volleyball players often complete ~70 jumps per match [29,30]. Although youth athletes may accumulate fewer total jumps, the repeated exposure across training and competition likely contributes to these differences. Collectively, frequent practice of explosive sport-specific movements likely improves jumping technique, which may explain why athletes with more years of formal participation were more often classified in the high-performance clusters [31,32].

In contrast, training experience did not distinguish between the two groups for maximal torque (Figure 2), torque steadiness (Figure 3), or postural control (Figure 4). However, years of sport participation provide a coarse measure of exposure and may not reflect the specific stimulus required to improve these attributes. For instance, [33] athletes tend to exhibit superior static balance compared to control individuals, indicating that engagement in sport enhances postural control. Furthermore, the present findings contrast with previous reports from sports that place substantial demands on dynamic stability, such as gymnastics [26], suggesting that sport-specific demands, rather than general experience, determine adaptation in these domains. Similarly, team-sport athletes exhibit greater lower-limb power output and maximal strength compared with controls, underscoring the specificity in the adaptations [34,35]. These adaptations are likely explained by structural differences (e.g., muscle volume) that develop in athletes compared with non-athletes [36,37]. Presumably, similar differences may exist between more and less experienced athletes, analogous to those reported between athletes and non-athletes, as cumulative exposure to sport alone may be insufficient to elicit task-specific adaptations in less experienced individuals.

### 4.3. Overlap in Maturity Status and Training Experience Between Performance Groups

Despite differences between groups, the influence of maturity offset and training experience overlapped between high- and low-performers across most tasks. This indicates that neither maturity nor training exposure was independently related to performance. One interpretation is that maturation influences the probability of achieving higher performance scores without guaranteeing it. The variability between individuals in maturation timing, responsiveness to training, and sport-specific exposure may allow some less mature athletes to perform at levels comparable to their more mature peers, and conversely, some more mature athletes may not exhibit superior performance (Figure 1 and Figure 2). Overall, these findings suggest that maturity status—as assessed with an index based on peak height velocity—provides some useful information about the performance capabilities of youth (9–17 years) athletes.

### 4.4. Limitations

The present findings should be interpreted in light of several limitations. First, because k-means clustering classified participants into two groups based on each of the four performance metrics, the observed differences should be interpreted as exploratory associations rather than causal relationships. Second, training experience was quantified as years of participation and did not capture training intensity. Additionally, chronological age, maturity offset, and experience were interrelated, limiting the isolation of independent effects. Moreover, current results are constrained by the small cluster size for some tasks (e.g., jumps) that presumably reduced statistical power. The heterogeneous sport backgrounds of participants may have introduced task-specific adaptations, which could preferentially influence some outcomes, such as postural control, over others. Finally, although the potential role of sex differences is accommodated in the regression equations used to estimate maturity, a more thorough assessment of developmental status is necessary before it is possible to determine the influence of biological sex on the outcomes.

## 5. Conclusions

The more mature individuals—as assessed by peak height velocity—exhibited superior performance in assessments of torque steadiness and maximal strength of ankle plantar flexors and dorsiflexors. However, the performance of individuals with more sports experience was greater for powerful actions, such as the two vertical jumps. However, the influence of the two attributes was not consistent across the four performance metrics. Overall, these findings highlight the importance of monitoring maturity status and training experience along with other variables in youth athletes to guide athletic development. The adoption of task-specific classifications based on performance in technical, tactical, and physical demands of each sport would likely provide more informed coaching decisions and guidance in the development of youth athletes.

## Figures and Tables

**Table 1:** Mean ± SD for body weight, age, height and training experience for males (n = 61) and females (n = 23).

Sex	Body Weight (kg)	Age (yrs)	Height (cm)	Training Experience (yrs)
Males				
Mean	55.8	13.1	165	6.5
SD	15.1	1.7	12	2.1
Females				
Mean	60.8	13.7	166	6.7
SD	11.1	1.9	10	2.2

## Data Availability

Data and custom scripts will be made available on reasonable request.

## Abbreviations

The following abbreviations are used in this manuscript:

CMJ	CounterMovement Jump
SJ	Squat Jump
CoV	Coefficient of Variation
CoP Vel	Center of Press
MVC	Maximal Voluntary Contraction
